# Therapeutic efficacy and pharmacokinetics of liposomal-cannabidiol injection: a pilot clinical study in dogs with naturally-occurring osteoarthritis

**DOI:** 10.3389/fvets.2023.1224452

**Published:** 2023-08-23

**Authors:** Yael Shilo-Benjamini, Eran Lavy, Nadav Yair, Joshua Milgram, Daniel Zilbersheid, Atara Hod, Dinorah Barasch, Wiessam Abu Ahmad, Ahuva Cern, Yechezkel Barenholz

**Affiliations:** ^1^Department of Biochemistry, Hadassah Medical School, Laboratory of Membrane and Liposome Research, The Hebrew University of Jerusalem, Jerusalem, Israel; ^2^Koret School of Veterinary Medicine, The Robert H. Smith Faculty of Agriculture, Food and Environment, The Hebrew University of Jerusalem, Rehovot, Israel; ^3^The Mass Spectrometry Unit, School of Pharmacy, The Hebrew University of Jerusalem, Jerusalem, Israel; ^4^Hadassah Medical Center, The Hebrew University of Jerusalem, Jerusalem, Israel

**Keywords:** analgesia, cannabidiol, CBD, dogs, liposomes, osteoarthritis, pharmacokinetics, prolonged release

## Abstract

**Introduction:**

Osteoarthritis is a common disease in dogs resulting in chronic pain and decreased wellbeing. Common analgesics such as non-steroidal anti-inflammatories may fail to control pain and can produce major adverse effects. Study objectives were to evaluate pharmacokinetics, therapeutic efficacy, and safety of subcutaneous liposomal-cannabidiol (CBD) as an additional analgesic therapy in dogs suffering from naturally-occurring osteoarthritis.

**Methods:**

Six such dogs were recruited following ethics approval and owner consent. Dogs were administered a single subcutaneous injection of 5 mg/kg liposomal-CBD. Plasma concentrations of CBD, blood work, activity monitoring collar data, wellbeing questionnaire (owners) and pain scoring (veterinarian) were performed at baseline and monitored up to six weeks following intervention. Data overtime were compared with baseline using linear-regression mixed-effects. *P*-value was set at 0.05.

**Results:**

CBD plasma concentrations were observed for 6 weeks; median (range) peak plasma concentration (C_max_) was 45.2 (17.8–72.5) ng/mL, time to C_max_ was 4 (2–14) days and half-life was 12.4 (7.7–42.6) days. Median (range) collar activity score was significantly increased on weeks 5–6; from 29 (17–34) to 34 (21–38). Scores of wellbeing and pain evaluations were significantly improved at 2–3 weeks; from 69 (52–78) to 53.5 (41–68), and from 7.5 (6–8) to 5.5 (5–7), respectively. The main adverse effect was minor local swelling for several days in 5/6 dogs.

**Conclusion:**

Liposomal-CBD administered subcutaneously produced detectable CBD plasma concentrations for 6 weeks with minimal side effects and demonstrated reduced pain and increased wellbeing as part of multimodal pain management in dogs suffering from osteoarthritis. Further placebo-controlled studies are of interest.

## 1. Introduction

Osteoarthritis is one of the prevalent diseases in geriatric dogs, which usually results in chronic pain and decrease or loss of function (1–3). Conservative management of canine osteoarthritis uses long term non-steroidal anti-inflammatory drugs (NSAIDs) in order to reduce inflammation and control pain (4–7). However, NSAIDs may not be sufficient to control pain and their long-term use can be associated with gastrointestinal, hepatic, and renal adverse effects (7–9).

Cannabidiol (CBD) and tetrahydrocannabinol (THC) are the primary derivatives of the plant *Cannabis sativa*. While THC is highly psychoactive and may result in neurological signs in dogs (10, 11), CBD has no psychoactive activity and can be administered safely at high doses or for long periods (10, 12, 13). CBD was reported to alleviate chronic pain in people (14–16), and recently its effectiveness was reported in dogs with osteoarthritis (17–20). The recommended route of administration is orally with a frequency of twice daily (17, 19). In people, the bioavailability of CBD is considered to be as low as 6% (21). In dogs, bioavailability may be better, although, depending on the formulation and the dose used, plasma levels may be variable between studies and within a study between individual dogs (13, 17, 20, 22, 23). Another concern with oral oil-based CBD preparations is the palatability of the preparation, which may decrease dog compliance to the treatment (24).

Alternative, injectable route of CBD delivery using liposomes was reported recently (25). Liposomes are vesicles made of one or more bilayers of well-characterized phospholipids. They are attractive for pharmaceutical application because this delivery system is biocompatible, biodegradable, and non-toxic (26–28). Additionally, the US Food and Drug Administration (FDA) has approved many liposomal drug-products (28). Prolonged-release injectable liposomal-CBD formulation allows a more convenient administration route with better pet and owner compliance, and with the potential to increase CBD bioavailability (25).

The objectives of this pilot study were to evaluate the pharmacokinetics, therapeutic efficacy and safety of a single subcutaneous injection of liposomal-CBD using synthetic CBD in dogs with naturally-occurring osteoarthritis. Our hypotheses were that CBD will be detected for several weeks, there will be an improvement in dogs’ activity, pain level and wellbeing without major adverse effects.

## 2. Methods

### 2.1. Animals

The study was approved by the Institutional Animal Care and Use Committee (IACUC; approval protocol MD-21-16,661-2), and a signed informed consent was obtained from all dog owners or legal guardians. Six dogs suffering from naturally-occurring osteoarthritis at least in one joint were recruited to this study. Following an orthopedic examination, osteoarthritis was confirmed radiographically, and complete blood count and biochemistry panel were performed before initiation of the study. Exclusion criteria included dogs that were younger than 2-or older than 15-years, orthopedic surgeon recommendation for any joint surgery, undergoing a surgical procedure 3 months before intervention, or suspected liver disease. For ethical reasons, all dogs continued receiving analgesics and joint supplements that were prescribed prior to recruitment.

### 2.2. Liposomal-CBD intervention

Liposomal-CBD formulation (CBD Liposome Platform Technology; LPT) was obtained from Innocan Pharma^™^ (Israel). According to the product certificate of analysis, the Liposomal-CBD was prepared under strict aseptic conditions. Prior to use samples were submitted to Hy-Labs (Rehovot, Israel), a certified and accredited laboratory by the Israeli Ministry of Health and FDA, to confirm the formulation was sterile and below the approved limit of endotoxins. The results of these tests met the requirements of extra-vascular administered drugs in people.

The liposomal-CBD formulation was composed of synthetic CBD (Purisys LLC., Athens, GA, United States; not considered a controlled substance) that was loaded at a concentration of 50 mg/mL into hydrogenated soy phosphatidylcholine (HSPC) liposomes (Lipoid GmbH, Ludwigshafen, Germany).

The injection was performed between the shoulders, after hair clipping and aseptic skin preparation. Liposomal-CBD was injected subcutaneously at a dose of 5 mg/kg (0.1 mL/kg) using a 21-gauge, 1-inch needle at the prepared skin area.

### 2.3. Monitoring

#### 2.3.1. Pharmacokinetics

One mL blood was collected from a peripheral vein (cephalic or saphenous) for pharmacokinetic analysis at 2 and 6 h, 1, 2 and 4 days, and weekly 1–6 weeks following injection. Blood was collected into ethylenediamine tetra-acetic acid (EDTA) 1 mL tubes and centrifuged to separate the plasma within 5 min from collection. Plasma was immediately frozen at −20°C and then kept at −80°C until analysis. CBD quantification was performed using UHPLC-tandem mass spectrometry (LC–MS/MS) method, which was reported by the authors recently, and can be found in the Supplementary material at: https://www.frontiersin.org/articles/10.3389/fvets.2022.892306/full#supplementary-material (25). Pharmacokinetic parameters were calculated for 6 weeks following injection using a non-compartmental analysis with Phoenix WinNonlin (Certara^™^, NJ, United States, Version 6.3).

In 3/6 dogs an intravenous catheter was placed in the cephalic vein and left in place for 24–48 h to facilitate blood sampling.

#### 2.3.2. Pain assessment

The Canine Brief Pain Inventory (CBPI) (29, 30) was used as an owner questionnaire assessment. Briefly, this questionnaire includes a pain (scale 0–40) and function (scale 0–60) assessments, summed to a total scale of 0–100, where 0 = normally functioning dog with no pain, and 100 = non-functioning dog with worse possible pain. In addition, an overall CBPI quality of life assessment is given using a descriptive scale: poor, fair, good, very good and excellent. A pain interactive visual analog scale (iVAS) was used for veterinary assessment with a scale of 0–10; 0 = no pain, 10 = worse possible pain. Both assessments were completed at baseline before injection and then once weekly up to 6 weeks from injection.

#### 2.3.3. Activity monitoring collar and vital signs

At least two weeks before intervention, an activity monitoring collar (PetPace, Burlington, MA, United States^1^) (31, 32) was placed on the dogs’ neck. Data was collected from the collar for 2 weeks prior and 6 weeks following liposomal-CBD injection. For each dog the mean weekly score of four parameters (activity score, position score, calories expedite and sleep score) was obtained from the PetPace platform and analyzed for all dogs after completion of the study.

Physiologic parameters were monitored throughout the study period: heart rate (HR) using a stethoscope, respiratory frequency (*f*_R_) by observing thoracic excursions, rectal temperature (RT) via digital thermometer, and mean arterial blood pressure using an oscillometric blood pressure monitor (CASMED 740; CAS Medical Systems Inc., Branford, CT, United States) with the cuff placed above the carpus over the radial artery while the dog was in sternal recumbency. The physiologic parameters were measured at baseline and then at 2 and 6 h, 1, 2 and 4 days, and weekly 1–6 weeks following injection.

#### 2.3.4. Blood work

Blood samples (1–1.5 mL) were collected in EDTA tubes for complete blood count (CBC; ADVIA 2120i Hematology System, Siemens Healthineers, Erlangen, Germany; including clinical pathology assessment of blood smears) and in tubes containing a separator gel (CAT Serum Sep Clot Activator, Vacuette^®^, Greiner Bio-One, Kremsmünster, Austria; 2–2.5 mL) for biochemistry panel (cobas^®^ 6,000, Roche Diagnostics Corporation, Indianapolis, IN, United States) at baseline and then at 1 and 4 weeks after intervention. In two of the dogs, additional blood work was performed 2 days following injection.

#### 2.3.5. Adverse effects and follow-up

During the 6 weeks after injection, dogs were monitored closely for adverse effects; at the hospital during the first 6 h after injection, by the veterinarian at each time-point of blood sampling for PK, and by the owners at home throughout the 6 weeks. Following study termination, dog owners were contacted by phone once monthly for 6 more months, and then every 3–4 months. Additionally, owners were requested to inform the attending veterinarian of any change in health status of their dog.

### 2.4. Statistical analysis

Power analysis was not performed, as due to safety reasons the number of participants was limited to 6 dogs by the IACUC. Statistical analysis was performed using Stata/SE statistical software version 15.0 (StataCorp, College Station, TX, United States). Because sample size was small, descriptive statistics are expressed as median (range as minimum-maximum). Data analysis was performed with repeated measures mixed-effects with random intercept at the dog level. All values at time points following intervention were compared with baseline. Additionally, the association between CBD plasma concentrations and CBPI and iVAS scores were tested using mixed-effects linear regression. A *p*-value <0.05 was considered significant.

## 3. Results

### 3.1. Animals

Three spayed female and three male (1 neutered, 2 intact) dogs with a median age of 12 (9–14) years old and body weight of 34 (26–58) kg were recruited to the study and completed the 6 weeks monitoring period. Dogs’ signalments, joints affected, osteoarthritic supplements and routine oral analgesics are presented in Table 1.

**Table 1:** Data of 6 dogs suffering from osteoarthritis that were administered a single subcutaneous liposomal-cannabidiol (CBD) injection in addition to routine analgesic treatments.

Dog	Sex	Age (years)	Breed	Body weight (kg)	Affected joints	Supplements and analgesics	Other health conditions
1	Spayed female	14	Samoyed	26	Hip	Glucosamine, hyaluronic acid, chondroitin sulfate	
2	Neutered male	12	Mixed	58	Hip	Glucosamine, gabapentin	Kidney disease, suspected lumbar partial disc herniation
3	Spayed female	12	Mixed	28	Hip, stifles, and shoulders	Occasional previcox	
4	Spayed female	9	Mixed	36	Hip, stifles, and shoulders	Curcumin (turmeric), dipyrone	Kidney disease, lumbar pain
5	Male	14	Flat-coated retriever	36	Hip, stifles, and left shoulder	Glucosamine, occasional dipyrone	
6	Male	12	Malinois	32	Hip, stifles, tarsus, shoulders, elbows, carpus	Glucosamine, gabapentin, previcox	

### 3.2. Pharmacokinetic data

**Figure 1:**
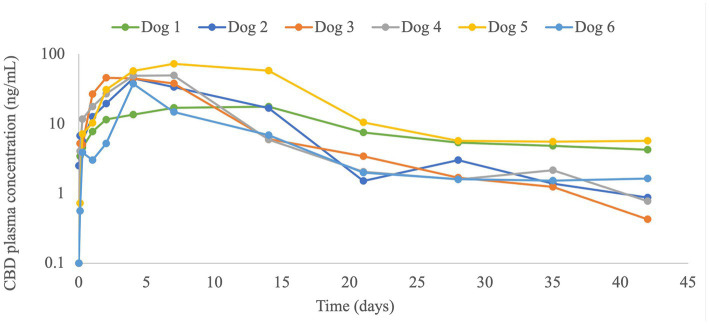
Plasma cannabidiol (CBD) concentrations (ng/mL) in 6 dogs with osteoarthritis before and up to 42 days (6 weeks) after a single subcutaneous liposomal-CBD injection at 5 mg/kg.

CBD plasma concentrations were observed throughout the 6-weeks monitoring period, including at the 6-week time point (Figure 1; Table 2). The plasma profile obtained showed a gradual increase in CBD up to the maximal CBD plasma concentration (C_max_), and then a decrease starting in most dogs (4/6) at one week following injection. In dog number 1 the increase and the decrease were very gradual, and in dog number 6 the decline started earlier, after 2-days from injection (Figure 1). Calculated pharmacokinetic data and CBD plasma concentrations at 3- and 6-weeks following injection are presented in Table 2.

**Table 2:** Pharmacokinetic data of plasma cannabidiol (CBD) from six dogs with osteoarthritis after a single subcutaneous 5 mg/kg liposomal-CBD injection.

Dog	C_max_ (ng/mL)	C_21 days_ (ng/mL)	C_42 days_ (ng/mL)	T_max_ (days)	Half-life (days)	AUC (ng·h/mL)	AUC/dose (ng·h/mL/mg/kg)
1	17.8	7.5	4.3	14	42.6	9,810	1962
2	44.8	1.5	0.9	4	10.5	11,877	2,375
3	45.7	3.4	0.4	2	7.7	11,630	2,326
4	49.4	2.1	0.8	7	12.1^#^	12,380	2,476
5	72.5	5.7	5.9	4	12.8^#^	19,275	3,855
6	37.7	1.6	2.0	2	14.7^#^	4,529	906
Median	**45.2**	**2.8**	**1.5**	**4**	**12.4**	**11,754**	**2,351**

^#^Lambda < 0.8. C_max_, peak plasma concentration; C_21/42 days_, plasma concentration at 21/42 days from injection; T_max_, time to maximum plasma concentration; AUC, area under the concentration–time curve. Blood samples were collected for 6 weeks following injection. The bold values are the median of the 6 dogs.

### 3.3. Pain scores

Dogs had significantly improved CBPI pain scores compared with baseline at weeks 2–3 (*p* = 0.011 and 0.031, respectively), improved CBPI function scores at weeks 2 and 6 (*p* = 0.004 and 0.026, respectively), improved CBPI total scores at weeks 2–3 (*p* = 0.001 and 0.028, respectively) and borderline improvement at week 6 (*p* = 0.052), and improved CBPI quality of life at weeks 2–3 (*p* = 0.046 for both weeks; Table 3). iVAS pain scores were significantly improved at 1–3 weeks (*p* < 0.001) and at 4 weeks following injection (*p* = 0.034; Table 3). The improvement in pain scores was significantly associated with the pharmacokinetic profile obtained; total CBPI at weeks 1–6 (*p* < 0.001 to *p* = 0.039, coefficients −0.249 to −4.399) and iVAS at weeks 1 (*p* = 0.008, coefficient −0.018), 2 and 3 (*p* < 0.001, coefficients −0.09 and −0.326, respectively).

**Table 3:** Scoring of canine brief pain inventory (CBPI; pain scale 0–40, function scale 0–60, total scale 0–100; 0 = no pain/normal function, 100 = worse pain/no function, and overall quality of life: poor, fair, good, very good and excellent) by owners, interactive visual analog scale (iVAS; scale 0–10; 0 = no pain, 10 = worse pain) by an anesthesiologist, and activity monitoring collar (PetPace) scores from six dogs with osteoarthritis, before and six weeks after liposomal-cannabidiol (CBD) subcutaneous injection.

	Baseline	Week 1	Week 2	Week 3	Week 4	Week 5	Week 6
**CBPI**							
Pain	22 (17–32)	19.5 (17–28)	16.5 (14–28)*	18.5 (12–28)*	22.5 (19–28)	22.5 (18–33)	22.5 (15–31)
Function	44 (39–52)	41 (26–45)	36 (26–43)*	40 (26–54)	41 (30–49)	40 (20–55)	36 (18–49)*
**Total**	69 (62–78)	63 (43–68)	53.5 (41–68)*	57 (44–79)*	66 (50–72)	63.5 (40–82)	58 (33–80)
**CBPI Quality of life**	Fair (Fair-Good)	Good (Fair-Good)	Good (Fair-Very good)*	Good (Fair-Very good)*	Good (Fair-Good)	Fair-Good (Fair-Good)	Fair-Good (Fair-Good)
**iVAS**	7.5 (6–8)	6.5 (6–7)*	6.0 (5–7)*	5.5 (5–7)*	6.5 (6–8)*	7.0 (6–8)	7.5 (6–8)
**PetPace**							
Activity score	29 (17–34)	30 (17–38)	30 (20–37)	32 (21–36)	30 (20–43)	33 (21–42)*	34 (21–38)*
Position score	651 (552–820)	698 (527–937)	684 (495–766)	669 (559–791)	693 (533–872)	655 (587–837)	692 (539–903)
Calories expedite	1, 308 (1,009–1878)	1,311 (1,018–1735)	1,334 (1,041–1889)	1,327 (1,056-1,912)	1,303 (993–1,938)	1,318 (963–1,930)	1,309 (1,012-1,732)
Sleep score	83 (73–87)	83 (77–88)	83 (79–88)	82 (78–87)	81 (77–88)	83 (78–88)	84 (79–88)

Data is presented as median (range; minimum-maximum). *Significantly improved from baseline value (*p* < 0.05).

### 3.4. Activity monitoring collar and physiologic parameters

Collar activity scores were significantly increased on weeks 5–6 (*p* = 0.012 and 0.027, respectively). Position scores, calories expedite, and sleep scores did not change significantly from baseline recordings (Table 3).

HR decreased significantly from baseline at 6 h (*p* = 0.010), 4 days (*p* = 0.010), 4 weeks (*p* = 0.022), and 6 weeks from injection (*p* = 0.017). *f*_R_ decreased significantly from baseline at 2 h (*p* = 0.018), 2 and 4 days (*p* = 0.005–0.008), and 5–6 weeks (*p* = 0.003–0.008). MAP decreased significantly from baseline at 4 days from injection (*p* = 0.048), and no difference was observed in RT throughout the study period (Table 4).

**Table 4:** Physiologic parameters from six dogs with osteoarthritis, before and six weeks after liposomal-cannabidiol (CBD) subcutaneous injection.

Time	HR (bpm)	*f*_R_ (rpm)	RT (°C)	MAP (mmHg)
Baseline	114 (100–126)	24 (24–36)	38.4 (37.8–38.7)	110 (83–125)
2 h	102 (80–120)	20 (16–28)*	38.3 (37.6–38.7)	103 (89–116)
6 h	98 (64–116)*	24 (12–28)	38.4 (37.9–38.7)	109 (101–120)
1 day	116 (88–120)	22 (20–32)	38.3 (38.0–40.0)	102 (99–122)
2 days	104 (84–160)	20 (16–32)*	38.4 (38.0–39.6)	101 (95–120)
4 days	94 (84–112)*	20 (12–32)*	38.2 (37.8–38.7)	95 (89–111)*
1 week	100 (88–112)	24 (16–28)	38.0 (37.5–38.5)	104 (92–111)
2 weeks	100 (80–112)	26 (20–32)	38.1 (37.9–38.4)	103 (97–121)
3 weeks	102 (88–116)	24 (16–32)	38.3 (37.9–38.4)	104 (97–120)
4 weeks	94 (80–116)*	24 (16–32)	38.3 (37.9–38.5)	104 (95–123)
5 weeks	104 (84–112)	20 (20–24)*	38.0 (37.8–38.8)	100 (89–108)
6 weeks	94 (76–120)*	20 (16–24)*	38.3 (38.0–38.6)	104 (97–116)

Data is presented as median (range; minimum-maximum). HR, heart rate; f_R_, respiratory frequency; RT, rectal temperature; MAP, mean arterial pressure; bpm, beats per minute; rpm, respirations per minute. *Significantly different from baseline value (*p* < 0.05).

### 3.5. Blood work

Most median hematology and biochemistry values were within reference ranges at all measurement times, although, some parameters were changed significantly from baseline. White blood cells (WBCs), neutrophils and monocytes increased significantly from baseline at 2 days from injection (*p* < 0.001). These increases were mainly attributed to a dog that developed phlebitis around the intravenous catheter. At 1 week after injection WBCs increased in 3/6 dogs (in the reference range) and decreased in 1/6 dogs with no overall significant change. Eosinophils increased significantly at 4 weeks (*p* = 0.009). At 1 week, a significant decrease was observed in hematocrit (*p* = 0.046), packed cell volume (PCV; *p* = 0.006), mean corpuscular volume (MCV; *p* = 0.017) and reticulocytes (*p* = 0.031). Platelets decreased significantly at 2 days (*p* = 0.046) and increased significantly at 1 week (*p* < 0.001), and plateletcrit increased significantly at 1 week (*p* = 0.007; Table 5).

**Table 5:** Complete blood count and biochemistry panel performed in six dogs with osteoarthritis, before and after a single liposomal-cannabidiol (CBD) subcutaneous injection at 5 mg/kg.

Parameter	Reference range	Baseline	2 days (*n* = 2)	1 week	4 weeks
Hematology					
White blood cells (10^3^/μL)	5.2–13.9	8.2 (6.5–14.8)	14.1 (12.2–16.0)*	9.8 (7.5–11.7)	8.0 (6.6–10.6)
Neutrophils (10^3^/μL)	3.9–8.0	5.1 (4.4–11.6)	10.8 (9.8–11.9)*	6.9 (4.8–7.1)	5.3 (3.6–7.1)
Monocytes (10^3^/μL)	0.2–1.1	0.55 (0.36–0.65)	0.97 (0.65–1.29)*	0.61 (0.56–0.71)	0.51 (0.34–0.56)
Lymphocytes (10^3^/μL)	1.3–4.1	2.0 (1.0–2.7)	2.0 (1.6–2.4)	1.9 (1.3–3.4)	1. 9 (1.2–2.4)
Eosinophils (10^3^/μL)	0.0–0.6	0.37 (0.29–0.58)	0.13 (0.11–0.15)	0.45 (0.28–0.64)	0.46 (0.36–1.24)*
Basophils (10^3^/μL)	0.0–0.1	0.01 (0.0–0.04)	0.02 (0.01–0.02)	0.01 (0.0–0.02)	0.01 (0.0–0.02)
Neutrophils (%)	42.5–77.3	65.6 (60.9–78.7)	77.2 (74.5–79.9)*	69.4 (61.0–71.1)	64.7 (54.1–71.3)
Monocytes (%)	3.3–10.3	5.8 (4.4–8.6)	6.7 (5.3–8.1)	6.7 (5.0–9.4)	5.8 (3.8–7.3)
Lymphocytes (%)	11.8–39.6	24.1 (12.6–27.6)	14.2 (13.3–15.1)	18.8 (16.8–29.2)	21.8 (16.2–29.3)
Eosinophils (%)	0.0–7.0	4.7 (0.3–6.4)	0.9 (0.9)	4.2 (2.8–6.6)	6.1 (4.7–11.6)*
Basophils (%)	0.0–1.3	0.1 (0.0–0.4)	0.1 (0.1)	0.1 (0.1–0.2)	0.1 (0.0–0.2)
Red blood cells (10^6^/μL)	5.7–8.8	6.3 (4.6–6.7)	6.1 (5.5–6.6)	5.8 (4.6–6.5)	6.1 (5.8–6.8)
Hematocrit (%)	37.1–57.0	43.9 (35.1–51.3)	43.9 (38.9–48.8)	40.0 (34.8–45.8)*	45.0 (39.7–48.8)
Hemoglobin (g/dL)	12.9–18.4	15.0 (12.0–17.4)	14.8 (13.4–16.1)	13.6 (12.5–15.6)	15.1 (14.0–16.1)
Mean corpuscular volume (MCV; fL)	58.8–71.2	71.0 (67.8–76.3)	72.0 (70.1–73.8)	70.2 (67.7–75.0)*	70.4 (68.3–75.5)
Mean corpuscular hemoglobin (MCH; pg)	20.5–24.2	24.3 (23.4–26.0)	24.3 (24.2–24.4)	23.9 (23.0–26.9)	24.0 (23.2–25.8)
Mean corpuscular hemoglobin concentration (MCHC; g/dL)	31.0–36.2	34.0 (33.5–35.3)	33.8 (33.0–34.5)	34.3 (33.1–35.8)	33.9 (32.9–35.3)
Reticulocytes (10^9^/L)	0.0–60.0	91.9 (14.0–235.7)	71.3 (44.8–97.7)	52.2 (33.6–129.3)*	69.1 (27.7–183.4)
Reticulocytes (%)	0.0–1.5	1.5 (0.3–3.5)	1.1 (0.8–1.5)	0.9 (0.6–2.0)*	1.2 (0.5–2.8)
Platelets (10^3^/μL)	143–400	362 (242–495)	297 (253–340)*	437 (383–641)*	360 (312–449)
Plateletcrit (%)	0.1–0.4	0.43 (0.26–0.50)	0.32 (0.31–0.32)	0.48 (0.44–0.61)*	0.37 (0.32–0.43)
Mean platelet volume (MPV; fL)	7.0–11.0	10.7 (10.0–12.4)	11.0 (9.2–12.8)	10.8 (8.9–13.1)	10.1 (9.0–11.5)
Platelets distribution width (PDW; %)	40.6–65.2	54.8 (48.5–60.7)	51.9 (46.7–57.1)	59.0 (42.8–63.3)	50.1 (43.2–59.0)
Packed Cell Volume (PCV; %)		44 (35–54)	44 (38–49)	38 (35–44)*	42 (38–45)
Total solids (TS)		7.0 (6.0–9.0)	7.0 (6.5–7.4)	7.0 (6.2–8.2)	7.0 (6.8–8.4)
Biochemistry					
Creatine phosphokinase (CPK; IU/L)	51–399	152 (83–264)	80 (67–92)	120 (106–425)	122 (74–196)
Aspartate aminotransferase (AST; IU/L)	19–42	27 (24–30)	20 (19–21)	31 (18–40)	30 (20–45)
Alanine transaminase (ALT; IU/L)	19–67	65 (26–183)	79 (56–102)	51 (21–112)	95 (33–200)
Alkaline phosphatase (ALP; IU/L)	21–170	71 (29–874)	750 (737–762)	107 (34–701)	95 (29–1,005)
Gamma-glutamyl transferase (GGT; IU/L)	0–6	5 (3–9)	6 (5–6)	3 (3)*	3 (3–6)*
Amylase (U/L)	103–1,510	985 (673–1,994)	1,829 (1,114-2,543)	1,031 (630–1,612)	836 (607–1,709)
Triglyceride (mg/dL)	19–133	74 (45–410)	219 (96–342)	128 (52–246)	151 (82–280)
Cholesterol (mg/dL)	135–361	237 (165–371)	285 (227–343)	278 (161–358)	278 (169–409)
Total bilirubin (mg/dL)	0.0–0.2	0.15 (0.15)	0.19 (0.15–0.23)*	0.15 (0.15)	0.15 (0.15)
Glucose (mg/dL)	64–123	84 (76–96)	92 (89–94)	87 (66–96)	83 (71–92)
Albumin (g/dL)	3.0–4.4	3.8 (3.0–5.3)	3.5 (3.0–3.9)	3.2 (2.9–3.6)*	3.3 (2.9–4.7)
Total protein (g/dL)	5.4–7.6	7.1 (6.1–8.7)	6.2 (5.6–6.7)*	6.5 (6.0–8.0)*	6.7 (6.0–8.1)*
Urea (mg/dL)	10.7–53.5	31.1 (24.5–114.6)	26.7 (23.8–29.5)	26.3 (18.6–35.5)	30.5 (23.4–58.2)
Creatinine (mg/dL)	0.3–1.2	1.02 (0.79–1.78)	0.85 (0.62–1.08)	0.84 (0.72–0.99)*	0.92 (0.68–1.13)
Phosphate (mg/dL)	3.0–6.2	3.78 (2.23–4.81)	3.76 (3.52–3.99)	4.12 (2.85–4.32)	3.86 (3.16–4.42)
Calcium (mg/dL)	9.7–11.5	10.5 (9.7–11.5)	9.6 (8.8–10.4)*	10.3 (9.6–10.8)	10.5 (10.0–11.0)
Sodium (mmol/L)	140–154	147 (137–149)	147 (142–151)	148 (145–152)	148 (135–150)
Chloride (mmol/L)	104–118	106 (103–115)	104 (102–107)	108 (104–110)	106 (103–109)
Potassium (mmol/L)	3.6–5.3	5.45 (4.28–6.07)	4.56 (4.48–4.64)*	5.67 (4.52–6.05)	5.29 (4.62–5.98)
CO_2_ (mmol/L)	16–26	19.7 (15.9–21.8)	18.2 (16.8–19.5)*	21.1 (19.3–23.7)*	20.6 (19.6–21.5)

*Significantly different from baseline value (*p* < 0.05). Data is presented as median (range; minimum-maximum).

Clinical pathology assessment of blood smears revealed mature non-toxic neutrophils at baseline in all dogs. A mild number of neutrophils became bands with mild toxic appearance in 3 different dogs: at 2 days (1 dog that developed phlebitis associated with intravenous catheter positioning), at 1 week (1 dog) and at 4 weeks (1 dog). Mild number of reactive monocytes was observed at baseline in 5/6 dogs, which were absent at the 4-week assessment in 4 dogs and sustained in one of these dogs. Mild–moderate number of atypical granular lymphocytes was observed at baseline and throughout the monitoring period in 5/6 dogs. Although none of the dogs was anemic, occasional polychromasia was observed in 5/6 dogs at baseline and at the following assessments. The dog that did not show polychromasia had mild poikilocytosis at baseline, then mild spherocytosis and mild poikilocytosis at 1-week, which were not observed on the 4-week assessment.

Alkaline phosphatase (ALP) did not change significantly during the study, however, one dog (dog number 5) showed high ALP value at baseline, which was further increased at the 4-week measurement. Another dog (dog number 4) had ALP elevation only at the 2-day measurement, during an elevated HR event. Gamma-glutamyltransferase (GGT) decreased significantly from baseline at 1 and 4 weeks from injection (*p* = 0.002 and 0.015, respectively). Total bilirubin increased significantly at 2 days (*p* < 0.001). Albumin decreased significantly at 1 week (*p* = 0.008). Total protein decreased significantly at all time points (*p* = 0.001, *p* < 0.01 and *p* = 0.004, respectively). Creatinine decreased significantly from baseline at 1 and 4 weeks from injection (*p* = 0.004 and 0.044, respectively). When dog 2, who had a kidney disease, was excluded from the creatinine analysis, creatinine was still decreased significantly at 1 week (*p* = 0.001). Calcium and potassium decreased significantly at 2 days (*p* < 0.001 and *p* = 0.010, respectively). CO_2_ decreased significantly at 2 days (*p* < 0.001) and increased significantly at 1 week (*p* = 0.010; Table 5).

### 3.6. Adverse effects and follow-up

Local response (minor, non-painful swelling at the injection site) was observed in 5/6 dogs. The swelling was resolved (i.e., absorbed completely) within 3–6 days following appearance without any treatment (Table 6). One dog had an increased HR to 140–160 beats per minute starting approximately 36 h after injection, which resolved without treatment a day later. An echocardiogram revealed sinus tachycardia. Another dog developed a fever, which was attributed to phlebitis around an intravenous catheter that was left for 24 h for blood sampling. The catheter was removed, oral antibiotics was initiated, and the fever was resolved within 12 h.

**Table 6:** Adverse effects and follow-up of six dogs with osteoarthritis after a single liposomal-cannabidiol (CBD) subcutaneous injection at 5 mg/kg.

Dog	Local response	Adverse effects	Follow-up
1	None observed	None	Pancreatitis 8 weeks after injection (medications were given with butter). Resolved after 2-day hospitalization. Died in her sleep 1 year and 2 weeks after injection.
2	Yes, at 2 days	None	Euthanasia due to deterioration in lumbar neurologic condition 7 months after injection
3	Yes, at 4 days	None	Deterioration in osteoarthritis. At the time of manuscript submission, 1 year and 7 months following injection
4	Yes, at 4 days	Increased heart rate at 1–2 days after injection	Generally doing well. At the time of manuscript submission, 1.5 years following injection
5	Yes, at 4 days	Fever 1 day after injection (caused by phlebitis), resolved within 12 h of antibiotics administration	Generally doing well. At the time of manuscript submission,1.5 years following injection
6	Yes, at 1 week	None	Gastric ulcers 8 weeks after injection (high dose of Previcox for a long period). Euthanasia due to deterioration in life quality 5 months after injection

Local response (swelling at injection site) was resolved within 3–6 days without any treatment.

At the time of manuscript submission, one of the dogs died naturally more than a year following injection at the age of 15 years, and two dogs were euthanized 5- and 7-months following injection due to deterioration in their disease condition (Table 6).

## 4. Discussion

### 4.1. Pharmacokinetics

Results from the present study suggest that a single subcutaneous liposomal-CBD administration provides long-term (i.e., several weeks) CBD plasma concentration and analgesia. Liposomal delivery systems provide a slow release of various encapsulated drugs (26, 28). Additionally, many liposomal-based formulations improve the therapeutic window of drugs and therefore reduce their toxicity (33). The use of liposomes as a delivery system for CBD in the present study, indeed provided slow drug release during the tested period, as was shown by the time it took to reach C_max_ (T_max_; 2–14 days) and by the long half-life (7.7–42.6 days) (Table 2). Compared with various oral CBD-containing formulations administering a single 2 mg/kg dose in dogs, the median/mean C_max_, T_max_, and half-life were 102.3 ng/mL, 1.5 h, and 4.2 h, respectively (*n* = 4) (17); 213 ng/mL, 2.1 h, and 2.5 h, respectively (*n* = 6) (11); 301 ng/mL, 1.4 h, and 1.0 h, respectively, (22); 226 ng/mL, 2.5 h, and 3.8 h, respectively (34).

In people, bioavailability of CBD is very low (6%–10%) and depends on fasting conditions (21). In dogs, bioavailability is considered better than in people, and reported to be 13%–70% depending on the formulation used (23, 35, 36). First-pass liver metabolism is believed to be the primary reason for the low bioavailability of oral CBD (21, 37). Therefore, alternative routes of delivery, such as via mucosal absorption that would bypass the liver are of interest. A recent study investigated the pharmacokinetics of a single 1 mg/kg pure CBD in oil formulation via oral transmucosal (OTM) administration or orally (6 dogs per route). Mean C_max_ and T_max_ for OTM and oral routes were 200.3 ng/mL and 1.9 h, and 206.8 ng/mL and 2.2 h, respectively. Half-life was 2.6 h with both routes (37). Interestingly, there was no difference in pharmacokinetic parameters between administration routes, suggesting that absorption via oral mucosa was not optimal or that most of the drug was actually swallowed (37). CBD administration was also investigated via nasal mucosa (mean dose of 1.7 mg/kg) or intrarectally using suppositories (mean dose of 8.3 mg/kg) compared with oral route (mean dose of 8.3 mg/kg). Following rectal administration CBD levels were below the limit of quantification. Mean C_max_ and T_max_ for nasal and oral routes were 28 ng/mL and 0.5 h, and 217 ng/mL and 3.5 h, respectively. Terminal elimination half-life was 7.0 and 15.7 h, respectively (38). According to these studies, CBD administered via mucosal sites was inferior compared with oral administration in dogs, although more studies using different CBD formulations are required for conclusion. This is strengthened by a study in dogs with naturally occurring osteoarthritis reporting a significant improvement following OTM CBD compared with control dogs (19).

Administration of Sativex^®^ (phytocannabinoid-based) sublingual spray was investigated in healthy young beagles, using an approximate dose of 0.5 mg/kg. Following a single dose, mean C_max_ and T_max_ of CBD were 10.5 ng/mL and 2 h, respectively (39). It should be noted that blood was sampled from the jugular vein in the Sativex^®^ study, which may have resulted in a biased overestimation of CBD plasma concentrations, because the jugular sampling site was reported to affect concentration of drugs administered via the oral mucosal route (40).

Transdermal administration was also investigated in two studies; (i) one study administered CBD-infused transdermal cream applied to the pinnae, which was compared with two oral formulations (CBD-infused oil or microencapsulated oil beads). These formulations were tested at 5 mg/kg twice daily in young healthy beagles (*n* = 10 per treatment). Following a single dose, mean C_max_ and half-life reached 625.3 ng/mL and 3.3 h (infused oil), 346.3 ng/mL and 1.6 h (oil beads), and 74.3 ng/mL (transdermal cream), respectively. The half-life of the CBD-infused transdermal cream could not be determined due to lack of elimination phase (23). (ii) The second study administered a transdermal low-THC *Cannabis sativa* extract 4 mg/kg rubbed into the pinnae twice daily for two weeks in six healthy young beagles. Mean C_max_ was 12.8 and 10.6 ng/mL after 7- and 14-days of administration. The authors concluded that CBD absorption via the transdermal route was generally poor (41).

In the present study C_max_ was lower compared with CBD plasma/serum concentrations at steady-state following 2–6 weeks oral CBD administration in dogs; 60–125 ng/mL (34), 80–160 ng/mL (23), 5–860 (median 311) ng/mL (20), and 53–201 ng/mL (12). This difference in C_max_ could be the effect of the relatively lower dose used with the prolong-release liposomal formulation, which was based on the reported dose tested intravenously (36). In retrospect a higher dose could have been tested. On the other hand, in many studies of oral CBD in dogs, C_max_ among individuals was extremely variable, with some dogs reaching only 10th of CBD plasma concentrations of other dogs in the same study using the same formulation (20, 24, 35, 37). Reduced variability among dogs in the present study suggests a more uniform drug absorption across dogs. Subcutaneous injected CBD has the benefit of direct absorption and bypassing the high extraction ratio of CBD by the liver compared with the oral route (21). Furthermore, when evaluating prolong-release formulations, the area under the curve (AUC) is the most important assessment tool, as it presents the total drug exposure over time (28). When normalized to dose, the AUC following liposomal-CBD administration in the present study (2,351 ng·h/mL/mg/kg; Table 2) was higher in comparison to long-term/steady-state oral CBD administration; 241–480 ng·h/ml/mg/kg after 28 days, once a day 1–12 mg/kg (12), 346–588 ng·h/ml/mg/kg after cannabis herbal extract containing 1:20 THC:CBD at 2–10 mg/kg (11), or 328–423 ng·h/ml/mg/kg after 2 mg/kg twice daily for 2 weeks of three different forms of hemp extract (34). Therefore, it suggests that the exposure to CBD using the liposomal formulation is superior to the oral route.

### 4.2. Pain and analgesia

CBD is known to have anti-inflammatory and anti-nociceptive effects (42–44) and was described in the past few years as an efficacious analgesic in dogs suffering from osteoarthritis (17–20). The therapeutic efficacy reported in the present study is similar to previous studies with pain reduction and improved function in all dogs. The endocannabinoid system plays an important role in afferent and efferent nociceptive pathways (45). CBD is considered to exhibit its anti-inflammatory properties and analgesia via cannabinoid receptor 2 (CB2) as an inverse agonist and as an inhibitor of the reuptake of the endocannabinoid anandamide (15, 45, 46). Additionally, CBD was reported to interact with many other receptors and channels that are involved in nociception, such as activation of serotonin receptors (5-HT_1A_), activation of transient receptor potential channels, vanilloid subfamily (TRPV1), inhibition of tumor necrosis factor-alpha (TNF-α), and inhibition of adenosine transporters (15, 45, 47). Furthermore, CB2 receptors expression is upregulated during inflammation in the affected tissue, as occurs in an osteoarthritic or rheumatoid joint. Therefore, treatment with cannabinoids activates CB2 receptors, and results in inhibition of cytokine production, decrease in leukocyte infiltration, reduction in bone destruction, and pain relief (45).

Unfortunately, the plasma CBD dose–response curve in dogs is still unknown. In the present study, a significant improvement in CBPI and iVAS pain scores was observed up to 3–4 weeks from injection, which corresponded to a median CBD concentration of 2.8 ng/mL. This may suggest that at this CBD plasma concentration there is still an analgesic effect, although, it is possible that the positive effect is also attributed to the overall high exposure observed.

### 4.3. Activity monitoring collar

Mobility in dogs can be affected by osteoarthritic pain, as previously reported (6, 32, 48). Therefore, the use of activity monitoring collars was chosen in order to provide an objective activity measurement. PetPace is a non-invasive monitoring collar that allows continuous monitoring of activity, position, certain vital signs, and sleep quality, and showed an excellent correlation with real-time variables (31, 49, 50). Recently, PetPace collar was suggested as a monitoring device to detect osteoarthritic pain, as it detected a significantly lower overall and high intensity activity levels in arthritic dogs when compared to healthy dogs (32). In the present study increased activity was observed 5–6 weeks following intervention, which was delayed from improvement in pain scoring evaluations, and CBD plasma levels. Factors other than pain can play a role in the pattern of dogs’ daily activity, such as owner activities, car rides, or environmental conditions (rain/extreme heat). Therefore, activity data from the collar, including data from the present study, should be interpreted with caution.

### 4.4. Blood work

Although some of the blood work values changed significantly from baseline during the monitoring period, most changes were not clinically important, as values were kept within the reference range. WBCs increased in some of the dogs, but were not above the reference range, except the dog who had phlebitis. The increase in WBCs can be explained by a mild response of the immune system to injection of foreign materials (51), and it suits the local response observed at the injection site. The authors are not aware of published studies evaluating the effect of other liposomal formulations on WBC count administered subcutaneously in dogs. Epidurally administered liposomal-morphine in dogs did not show a systemic elevation of WBCs, but WBC count in the CSF was higher in the liposomal-morphine group (17 ± 18 cells/mm^3^) versus the liposomal vehicle group (2 ± 1 cells/mm^3^), with a value of <20 as the normal range (52). Hematocrit decreased a week post injection, but it was mild with no clinical importance. ALP was reported to significantly increase from baseline following long-term (weeks to months) administration of oral CBD in dogs, which was thought to result from induction of liver CYP isoenzymes (22, 24). However, a recent study reported that increased ALP correlated with significant elevation in bone-specific ALP, suggesting that the rise in total ALP can be partly attributed to osteoblastic activity (13). In the present study ALP increased in two dogs (33%);one of them had increased levels at baseline, and the other dog had an increase only at the 2-day measurement. Albumin level decreased during the present study, although in the reference range. A recent study investigating long-term CBD administration in dogs reported that albumin decreased gradually and reached significant difference at 6-months from initiation of the CBD administration. But the albumin values were still within the reference range (13). Albumin level may be decreased due to effects on the liver, but no other changes related to liver function were observed. Other effects of the liposomal-CBD, such as proteinuria or inflammation, may have resulted in decreased albumin and should be further investigated.

### 4.5. Adverse effects

The minimal local swelling at the injection site was not diagnosed further, because it was minor, did not require a medical intervention, and was self-limiting. A different liposomal formulation (Exparel, DepoFoam Bupivacaine; made of phospholipids, cholesterol, and triglycerides) was reported previously to produce local response at the injection site in dogs. That study used experimental dogs and described the formation of granulomatous inflammation following multiple injections, characterized by an increased number of multinucleated giant cells and vacuolated macrophages. The authors of the Exparel study considered the local response as a normal response to the liposomes and non-adverse (51).

### 4.6. CBD drug-products in veterinary medicine

In recent years CBD has gained popularity in the veterinary market (13). However, products’ label can be misleading as many “CBD” products are actually extracts or enriched extracts from *Cannabis sativa*, and therefore they contain varying amounts of CBD in addition to many other chemically complex cannabis ingredients. A recent study reported that of 29 CBD products for dogs the total median CBD concentrations of their label claim was 93% (0%–154%) of claims (53). Valid CBD label-claims require rigorous analytical characterization and regulation (53). The FDA has published a guidance explaining that CBD products that are marketed without a prescription are not approved and may put users at risk (54, 55). Compared with cannabis-based products, synthetic CBD, which is FDA approved with a drug master file, provides a true THC and other cannabinoids-free product. The use of synthetic CBD as the active pharmaceutical ingredient of the liposomal-CBD formulation, can provide a reliable desired effect repeatedly.

### 4.7. Limitations

Limitations to this study include the small sample size, and the non-blinded study design, which could have introduced bias to the owner and veterinary evaluations. We calculated the bioavailability based on a study reporting intravenous CBD administration from 1988 (36), which may not be an accurate calculation, but no other study reporting intravenous CBD in dogs is available in the literature. Most of the dogs in this study were geriatric, which potentially can affect the absorption and elimination of the CBD, and younger animals may have different pharmacokinetic profile following liposomal-CBD. Although, this may also be a strength of this study, as some of the dogs had concurrent disease states and/or were receiving routine medications, and this is usually the population of dogs that can benefit from CBD treatment.

## 5. Conclusion

Liposomal-CBD administered subcutaneously had minor adverse effects, resulted in detectable CBD plasma concentrations for 6 weeks and showed high exposure in terms of AUC, which correlated with high bioavailability and decreased pain scores. This liposomal formulation can be used as an additional treatment as part of multimodal analgesia to increase wellbeing in dogs suffering from osteoarthritis. Further studies incorporating placebo-control, dose–response curve, and multiple injections (i.e., every several weeks) would provide more information as to the long-term efficacy and safety of this formulation.

## Data availability statement

The raw data supporting the conclusions of this article will be made available by the authors, without undue reservation.

## Ethics statement

The animal studies were approved by the Ein-Kerem Animal Care and Use Committee, The Hebrew University of Jerusalem. The studies were conducted in accordance with the local legislation and institutional requirements. Written informed consent was obtained from the owners for the participation of their animals in this study.

## Author contributions

YS-B and AC contributed to study conception, data acquisition and interpretation, and drafted the manuscript. NY, JM, DZ, AH, and DB contributed to the data acquisition. WA analyzed the data. EL and DB interpreted the results. YB contributed to study conception and interpreted the results. All authors contributed to the article and approved the submitted version.

## Funding

This study was funded by Innocan Pharma^™^.

## Conflict of interest

DZ, AH, and AC are supported by Innocan Pharma^™^. AC and YB have a patent pending on the liposomal-CBD formulation used in this study.

The remaining authors declare that the research was conducted in the absence of any commercial or financial relationships that could be construed as a potential conflict of interest.

## Publisher’s note

All claims expressed in this article are solely those of the authors and do not necessarily represent those of their affiliated organizations, or those of the publisher, the editors and the reviewers. Any product that may be evaluated in this article, or claim that may be made by its manufacturer, is not guaranteed or endorsed by the publisher.

## Footnotes

^1^ https://petpace.com/smart-sensing-collar/
